# Integrated network pharmacology to investigate the mechanism of *Salvia miltiorrhiza* Bunge in the treatment of myocardial infarction

**DOI:** 10.1111/jcmm.17932

**Published:** 2023-08-29

**Authors:** Xueying Huang, Muxin Zhang, Yu Song, Bowen Sun, Lin Lin, Xiaoli Song, Chao Li

**Affiliations:** ^1^ School of pharmacy Shandong University of Traditional Chinese Medicine Jinan China; ^2^ First Clinical Medical College Shandong University of Traditional Chinese Medicine Jinan China; ^3^ Innovative Institute of Chinese Medicine and Pharmacy Shandong University of Traditional Chinese Medicine Jinan China

**Keywords:** myocardial infarction, network pharmacology, *Salvia miltiorrhiza* Bunge, VEGF signalling pathway

## Abstract

*Salvia miltiorrhiza* Bunge is a natural drug for treating myocardial infarction (MI). However, the targets and mechanisms of *S. miltiorrhiza* Bunge in the treatment of MI are yet to be elucidated. Traditional Chinese medicine systems pharmacology (TCMSP) data were used to screen out chemical constituents, and UniProt was used to predict relevant targets. Disease targets were obtained from the Online Mendelian Inheritance in Man and GeneCards databases. We used the STRING platform to build a protein–protein interaction network and used Cytoscape_v3.8.1 software to make a Drug–Ingredients–Gene Symbols–Disease network map. The Metascape database was used to perform gene ontology and Kyoto Encyclopaedia of Genes and Genomes (KEGG) enrichment analyses for drug–disease overlapping gene symbols. The targets identified by network pharmacology were further verified by in vitro and in vivo experiments. Seventy‐five active components of *S. miltiorrhiza* Bunge were obtained from the TCMSP database, while 370 disease targets and 29 cross‐targets were obtained from the Genecards database. The KEGG pathway enrichment results suggested that the mechanism of *S. miltiorrhiza* Bunge in the treatment of MI was significantly related to the VEGF signalling pathway. In vitro and in vivo experiments were used to evaluate the reliability of some important active ingredients and targets. *S. miltiorrhiza* Bunge alleviated the damage to cardiac function, attenuated myocardial fibrosis and protected endothelial cell function by increasing the expression of TGF‐β and VEGFA. *S. miltiorrhiza* Bunge showed the therapeutic effect of MI by promoting the expression of VEGFA signalling pathway, providing a reliable basis for exploring herbal treatment of MI.

## 1 INTRODUCTION

According to the World Health Organization, myocardial infarction (MI) is the leading cause of mortality worldwide. The formation of plaques in the inner wall of arteries leads to a decrease in blood flow to the heart, resulting in a lack of oxygen supply to the myocardium and leading to myocardial damage, which is the pathogenesis of MI.^1^ Therefore, the key to MI treatment is to restore blood perfusion and alleviate myocardial ischaemia. In addition to basic medical therapy and thrombolysis, revascularization primarily relies on percutaneous coronary intervention (PCI) and coronary artery bypass grafting (CABG).^2^, ^3^ However, more than 30% of patients are not suitable for revascularization due to insufficient cardiac function,^4^ highlighting the need for a more effective and safer treatment strategies.

*Salvia miltiorrhiza* Bunge belongs to the Lamiaceae family, and its roots and rhizomes can be applied in a dry form. The main pharmacological active ingredients of *S. miltiorrhiza* Bunge are phenolic acids and tanshinone. Phenolic acids have a wide range of biological activities, including antioxidant, anticoagulant and anti‐inflammatory effects. Tanshinone is a fat‐soluble diterpene. Modern pharmacological research shows that tanshinone IIA (TanIIA) has anti‐inflammatory, anti‐myocardial ischaemia and anti‐atherosclerosis effects. In clinical practice, Tan IIA is widely used as an injection. Previous studies have reported that Tan IIA can promote the expression of angiogenic factors and upregulate Cx37, Cx40 and Cx43 to promote angiogenesis in endothelial cell‐like cells derived from mesenchymal stem cells.^5^ In addition, Tan IIA effectively blocks the abnormal activation of NF‐κB via TLR/NF‐κB pathway and MAPKs/NF‐κB pathway, thereby alleviates the inflammatory response after MI.^6^ However, the targets and mechanisms of *S. miltiorrhiza* Bunge in treating MI still need to be elucidated.

Network pharmacology methods can systematically determine the effects and mechanisms of drugs used to treat complex diseases at the molecular, cellular and biological levels.^7^ Therefore, we used network pharmacology to analyse drug targets and key pathways of *S. miltiorrhiza* Bunge in the treatment of MI. Additionally, both in vitro and in vivo experiments were performed to confirm the predictions synthesized by network pharmacology.

## 2 MATERIALS AND METHODS

### 2.1 Network pharmacology

#### 2.1.1 Screening of bioactive components of *Salvia miltiorrhiza* Bunge

All components of the Chinese medicinal herbs in *S. miltiorrhiza* Bunge were retrieved from the traditional Chinese medicine systems pharmacology (TCMSP) database (http://lsp.nwu.edu.cn/tcmsp.php). Preliminary screening of all components was conducted according to oral bioavailability (OB; ≥30%) and drug‐likeness (DL) properties (≥0.18) and supplement of all components according to published journals and literature.

#### 2.1.2 Prediction of drug targets for *Salvia miltiorrhiza* Bunge

The protein targets of the active substances in *S. miltiorrhiza* Bunge were retrieved from the UniProt database (https://www.uniprot.org/).

#### 2.1.3 Collection of gene targets for myocardial infarction

Myocardial infarction‐related human genes were collected from two databases. The first source was the GeneCards (http://www.genecards.org/). GeneCards is a searchable, comprehensive database that offers all comments and predicts human genetic information comprehensively in a user‐friendly manner. The second source was the Online Mendelian Inheritance in Man (OMIM) database (https://omim.org/).^8^

#### 2.1.4 Making Venn diagrams

Enter the active ingredient targets and MI targets of *S. miltiorrhiza* Bunge into the Venn diagram and apply online drawing tools (Venn 2.1.0, https://bioinfogp.cnb.csic.es/tools/venny/index.html) to get the Venn diagram.

#### 2.1.5 Construction of a Drug–Ingredients–Gene Symbols–Disease network

After obtaining the Venn diagram of overlapping gene symbols, we constructed a complex information network based on the interactions between drugs, ingredients, gene symbols and disease. The active ingredients of *S. miltiorrhiza* Bunge selected the components that intersect with the target of MI. Next, we used Cytoscape v3.8.1 (www.cytoscape.org/) to undertake visual analyses of the Drug–Ingredients–Gene Symbols–Disease (D–I–G–D) network,^9^ which is a graphical display of network analyses and editing software.

#### 2.1.6 Building protein–protein interaction network

The effective chemical constituents of *S. miltiorrhiza* Bunge from Venn diagram were imported into STRING database (https://string‐db.org/) to obtain the protein–protein interaction (PPI) network diagram and corresponding information.

#### 2.1.7 Gene ontology enrichment analysis and Kyoto Encyclopaedia of Genes and Genomes enrichment analysis

Using Metascape database (http://metascape.org/gp/index.html), is obtained by making Venn ‐ MI‐ *S. miltiorrhiza* Bunge effective chemical composition of intersection targets in the input to the database, then the intersection targets were analysed by gene ontology (GO) and Kyoto Encyclopaedia of Genes and Genomes (KEGG). The results were analysed using the microscopic letter online mapping platform (www.bioinformatics.com.cn/) online mapping tools to make the bubble chart display.

#### 2.1.8 Computational validation of ingredients–targets interactions

In order to ascertain the interaction between active ingredients and their protein and to examine their binding modes, we selected the active ingredients with the strongest binding and the top five targets with the median score of the PPI network for verification of molecular docking. The X‐ray crystal structures were obtained from the RCSB Protein Data Bank (PDB; www.rcsb.org). The drug molecular structures were downloaded from the PubChem database (https://pubchem.ncbi.nim.nih.gov/). Molecular docking calculations were performed using Autodock Vina 1.1.2, using Pymol 2.3.2 software to visualize the results.

### 2.2 Experimental validation

#### 2.2.1 The preparation of tanshinone IIA (TanIIA)

Tanshinone IIA (Shanghai standard biotechnology) was dissolved in dimethyl sulfoxide. According to the instructions, the solution was stored at −80°C in the dark.

#### 2.2.2 Cell culture

Human umbilical vein endothelial cells (HUVECs) were obtained from ScienCell Research Laboratories. Cells were cultured in endothelial cell medium (ECM). Cells were maintained at 37°C in a humidified chamber with 5% CO_2_. The control group was treated with DMEM containing 10% FBS and 1% penicillin and streptomycin (P/S); the oxygen‐glucose deprivation (OGD) group was added with sugar‐free DMEM medium containing 10% FBS and 1% P/S; and the Tan IIA group was added with 15 μg/mL TanIIA. The OGD group and Tan IIA group were maintained under a hypoxia incubator containing 2% oxygen after culturing for 24 h.

#### 2.2.3 Animals

Seven‐week‐old male mice with MI and normal C57BL/6J mice were both obtained from Vital River Laboratory Animal Technology Co. The study protocol was approved by the Ethics Committee of Shandong University of Traditional Chinese Medicine, and the animal experiments were performed in accordance with the Guide for the Care and Use of Laboratory Animals (published by the US National Institutes of Health). All mice were raised under SPF laboratory conditions with water and food at a temperature of 22 ± 2°C and kept on a 12‐h‐light/dark cycle throughout the experimental period. C57BL/6J mice (*n* = 8) were all used in the control group and injected intraperitoneally with 0.9% NaCl (2 mL kg^−1^) for 8 weeks. Left anterior descending (LAD) ligation surgery was performed on C57B/6 J mice, and post‐operative electrocardiogram measurements confirmed MI diagnosis. During the LAD procedure, a small incision was made on the sternum of the mice, followed by the separation of the sternum to expose the heart. The LAD was then ligated with a suture, causing restriction of blood supply to the mouse heart. After 3 days of being diagnosed with MI, the mice were divided into two groups on average, with one group receiving abdominal injection of 0.9% NaCl (2 mL kg^−1^) for 8 weeks (LAD group *n* = 8), while the other group receiving abdominal injection of TanIIA (15 mg kg^−1^) for 8 weeks (TanIIA group *n* = 8).

#### 2.2.4 Cardiogram assay

Mice were intraperitoneally injected with pentobarbital sodium and fixed onto wooden plates. Small metal syringes were inserted under the skin of the limbs of mice. The mouse electrocardiogram experiment was carried out using the RM6240E multichannel physiological signal acquisition system (Shanghai, China).

#### 2.2.5 Cardiac function analysis

Mice were intraperitoneally injected with pentobarbital sodium, and their limbs were fixed on a wooden board in the supine position, ensuring they were in a stable and calm state. Place the mice on a warm bed to maintain a stable body temperature, and ensure that the fur in the area being examined is clean and free of air bubbles. Ultrasound gel was applied to the ultrasound probe to improve ultrasound conduction and image quality. Place the ultrasound probe on the mouse's chest wall, positioning it over the heart area. M‐mode were used to observe heart wall motion and measure cardiac functional parameters. A small animal ultrasound instrument (Xuzhou) was used to detect the echocardiography.

#### 2.2.6 Assay for brain natriuretic peptide and N‐terminal pro‐brain natriuretic peptide

Brain natriuretic peptide (BNP) and N‐terminal pro‐brain natriuretic peptide (NT‐ProBNP) activity were measured using the Mouse BNP ELISA Kit and Mouse NT‐ProBNP ELISA Kit (Elabscience). Both the standard and serum samples from different treatment groups were added to the coated wells. Biotinylated detection Ab was then added and incubated at 37°C for 1 h. HRP conjugate was added and incubated at 37°C for 30 min. After adding the TMB solution and termination solution, the absorbance for the final product was measured at 450 nm. According to the standard curve OB value of the sample, the corresponding BNP and NT‐ProBNP were calculated.

#### 2.2.7 Pathological morphology analysis

Heart tissue was fixed with 3% glutaraldehyde fixation fluid (pH 7.2–7.4) for 24 h. The aortic sinus was sliced into 5 μm serial paraffin sections. Masson, Tunel and CD31 (Nanjing Jiancheng Technology Co., Ltd) were performed on the sections. The staining results were observed under a fluorescence microscope.

#### 2.2.8 Regional detection of ischaemic myocardium

Mice in different treatment groups were anaesthetized with pentobarbital sodium. The heart tissue was then taken and placed at −20°C for 30 min. The tissue was sliced into sections with a thickness of 2 mm. Staining was performed with haematoxylin and eosin. The staining results were observed under a microscope.

#### 2.2.9 Migration assay

The cell migration rate was determined using a Transwell assay chamber (Corning). HUVECs were diluted to 3.0 × 10^5^ mL^−1^ with serum‐free DMEM, and 200‐μL cell suspension was added to the upper compartment of the chamber. A volume of 600 μL DMEM medium supplemented with 10% FBS was added to the lower chamber and then incubated at 37°C for 6 h. The cells were fixed with 4% paraformaldehyde for 15 min and then stained with haematoxylin staining solution and eosin staining solution for 30 and 10 min, respectively. The cells were then counted under a microscope.

#### 2.2.10 Adhesion assay

Fibronectin was diluted with DMEM·F12 (1:9) and tiled in a 24‐well plate for 12 h at 4°C. After seeding the HUVECs into 24‐well plates, we placed them in a cell culture incubator containing 5% CO_2_ at 37°C for 4 h. Phosphate‐buffered saline was used to remove the unattached HUVECs. The cells were fixed with 4% paraformaldehyde for 10 min, then stained with haematoxylin staining solution and eosin staining solution for 30 and 10 min, respectively. The number of adherent cells was then counted under the microscope.

#### 2.2.11 Proliferative activity assay

According to the instructions of the EdU incorporation assay kit (Guangzhou, Ribobio), the EdU solution was diluted by ECM to 50 mM and incubated at 37°C for 4 h. Then 4% paraformaldehyde was added and incubated at room temperature for 30 min. An amount of 3 mg mL^−1^ of bovine serum albumin (BSA) was added to wash the cells in a decolorizing shaker for 5 min. Then the staining reaction solution (1×) was added to each well and incubated in dark for 30 min. Hoechst 33342 solution (1×) was then added and left to incubate in the dark for 10 min. The staining results were observed under a fluorescence microscope.

#### 2.2.12 Tube formation assay

Matrix glue (Matrigel) was added to the 96‐well plate and placed in an incubator with 5% CO_2_ at 37°C for 1 h. A volume of 100 μL of cell suspension was then added to each well (3 × 10^4^ per well). After 6 h, the number and integrity of tubular structures were observed. Representative pictures were taken under the microscope at 200×.

#### 2.2.13 Cell activity detection

Cell Counting Kit‐8 (CCK8; Meilunbio) was diluted 10 times according to instructions, incubated with cells with 5% CO_2_ at 37°C for 4 h, and absorbance was measured at 450 nm.

#### 2.2.14 Western blot analysis

The HUVECs and mice myocardial tissue samples were lysed with RIPA buffer. Protein concentrations were measured using a BCA protein assay kit (Interchim). Proteins were separated on a SDS polyacrylamide gel and were transferred onto PVDF membranes. After being blocked, the membranes were incubated with the following primary antibodies overnight at 4°C: VEGFA: (1:1000, 19003‐1‐ap, Proteintech), TGF‐β: (1:1000, 21898‐1‐ap, Proteintech) and β‐actin (1:1000, 23660‐1‐ap, Proteintech). Then the membranes were incubated with horseradish peroxidase‐conjugated secondary antibodies at room temperature for 1 h. The protein bands were detected using an ECL Substrate (Thermo Fisher Scientific), and the protein bands was performed using the ImageJ software.

#### 2.2.15 Statistical analysis

Data were analysed using Graphpad 7 software (GraphPad Software) and were expressed as the mean ± SD. Images were processed with ImageJ. *p* < 0.05 were considered statistically significant.

## 3 RESULTS

### 3.1 Network pharmacology analysis

#### 3.1.1 Screening of active ingredients and prediction of drug targets

After retrieval using the TCMSP database, a metabolic excretion screening of absorption distribution was carried out based on two parameters of oral bioavailability and drug similarity. A total of 65 active ingredients were obtained, which conformed to OB ≥ 30% and DL ≥ 0.18, and then we selected the candidate ingredients in *S. miltiorrhiza* Bunge intersect with the targets of MI; they were considered to be active ingredients. Sixty‐six active ingredients were selected from the TCMSP database and subsequently converted to the Uniprot database. After the duplicate items were deleted, a total of 90 targets were obtained. Four hundred twenty‐two disease targets were obtained from the OMIM and GeneCards databases. Two types of targets intersected, and 35 targets were discovered. Subsequently, corresponding active ingredients were selected from 35 targets, resulting in a total of 10 active ingredients. Detailed information was shown in Table 1.

**TABLE 1:** The top 10 active ingredients of *Salvia miltiorrhiza* Bunge.

No	MOL ID	Molecule name	Relative molecular mass	OB	DL
1	MOL000006	Luteolin	286.25	36.16	0.25
3	MOL001601	1,2,5,6‐tetrahydrotanshinone	280.34	38.75	0.36
3	MOL001659	Poriferasterol	412.77	43.83	0.76
4	MOL002651	Dihydrotanshinone IIA	292.35	43.76	0.4
5	MOL007041	2‐isopropyl‐8‐methylphenanthrene‐3,4‐dione	266.36	34.35	0.23
6	MOL007082	Danshenol A	336.41	56.97	0.52
7	MOL007101	Dihydrotanshinone I	278.32	45.04	0.36
8	MOL007130	Prolithospermic acid	314.31	64.37	0.31
9	MOL007145	Salviolone	268.38	31.72	0.24
10	MOL007154	Tanshinone IIA	294.37	49.89	0.4

Abbreviations: DL, drug‐likeness; OB, oral bioavailability.

After the initial collection of data from the TCMSP database, subsequent conversion into the UniProt database and the deletion of redundant items, a total of 90 targets were identified to obtain active ingredients.

#### 3.1.2 Acquisition of Gene Targets for myocardial infarction

The OMIM and GeneCards databases were used to search for MI‐related targets. A total of 422 disease targets were finally screened by removing duplicate target genes from the two databases.

#### 3.1.3 Intersection target of *Salvia miltiorrhiza* Bunge and myocardial infarction

**FIGURE 1:**
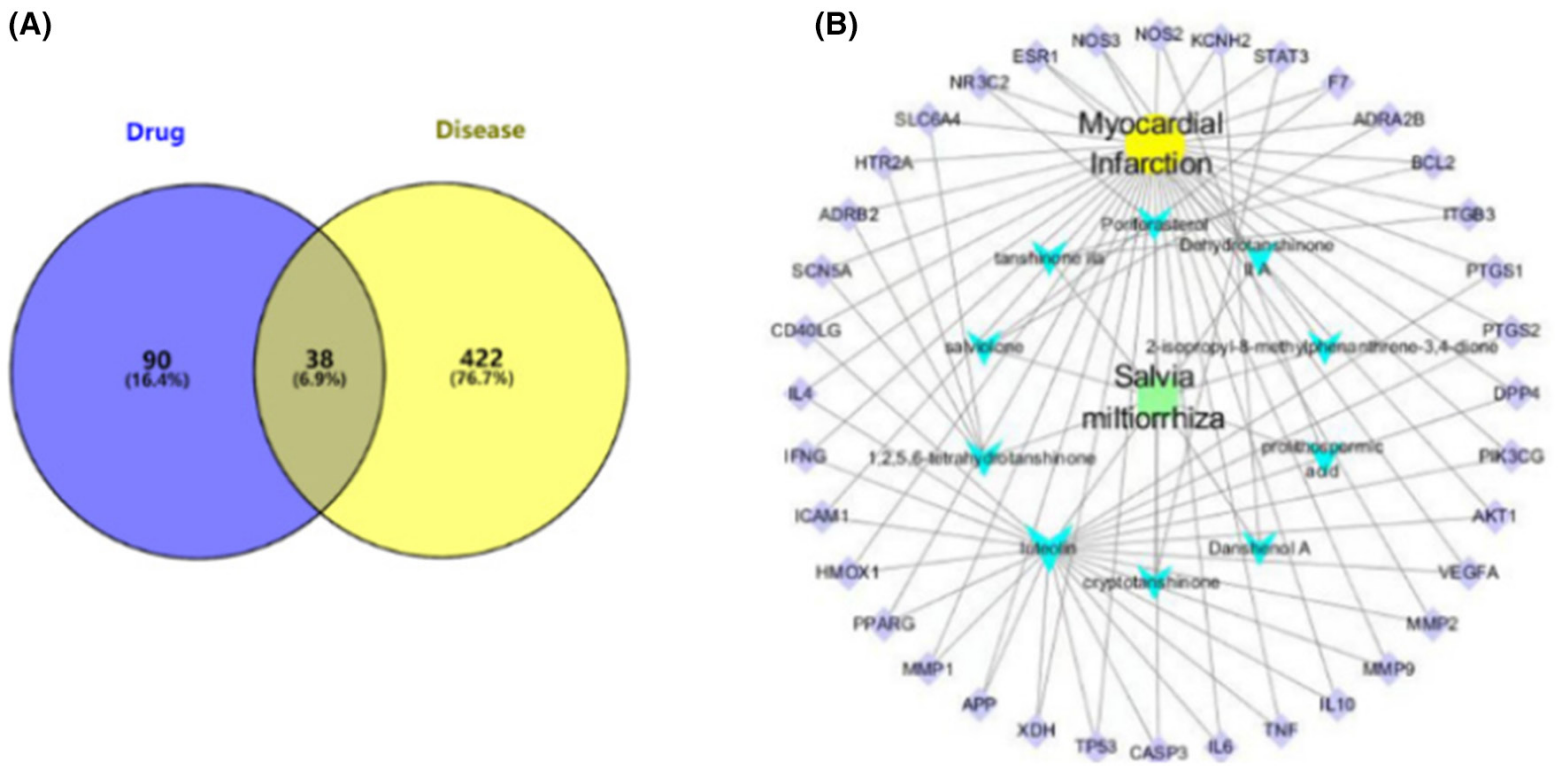
Venn and Drug–Ingredients–Gene Symbols–Disease (D–I–G–D) network. (A) 38 overlapping gene symbols between the disease and drug. (B) D–I–G–D network. The green node represents *Salvia miltiorrhiza* Bunge, and the yellow node represents MI. The 10 blue nodes represent the active ingredients in *S. miltiorrhiza* Bunge; The 38 purple nodes represent the overlapping gene symbols between the disease and drug. The edges denote hat nodes can interact with each other.

An online mapping tool was used to construct the Venn diagram of drug active ingredient targets and disease targets. Figure 1A showed that 38 of the 422 disease gene symbols and 90 drug–gene symbols were overlapping.

#### 3.1.4 Analysis of the Drug–Ingredients–Gene Symbols–Disease network

To clarify how *S. miltiorrhiza* Bunge may treat MI, a D–I–G–D network was built by Cytoscape (Figure 1B), whereby the green nodes represented *S. miltiorrhiza* Bunge and the yellow nodes represented MI. Additionally, the blue nodes represented the active ingredients in *S. miltiorrhiza* Bunge, while the purple nodes represented the overlapping gene symbols between drugs and the disease. Lines indicated the potential for nodes to interact.

#### 3.1.5 Analysis of the protein–protein interaction network

**FIGURE 2:**
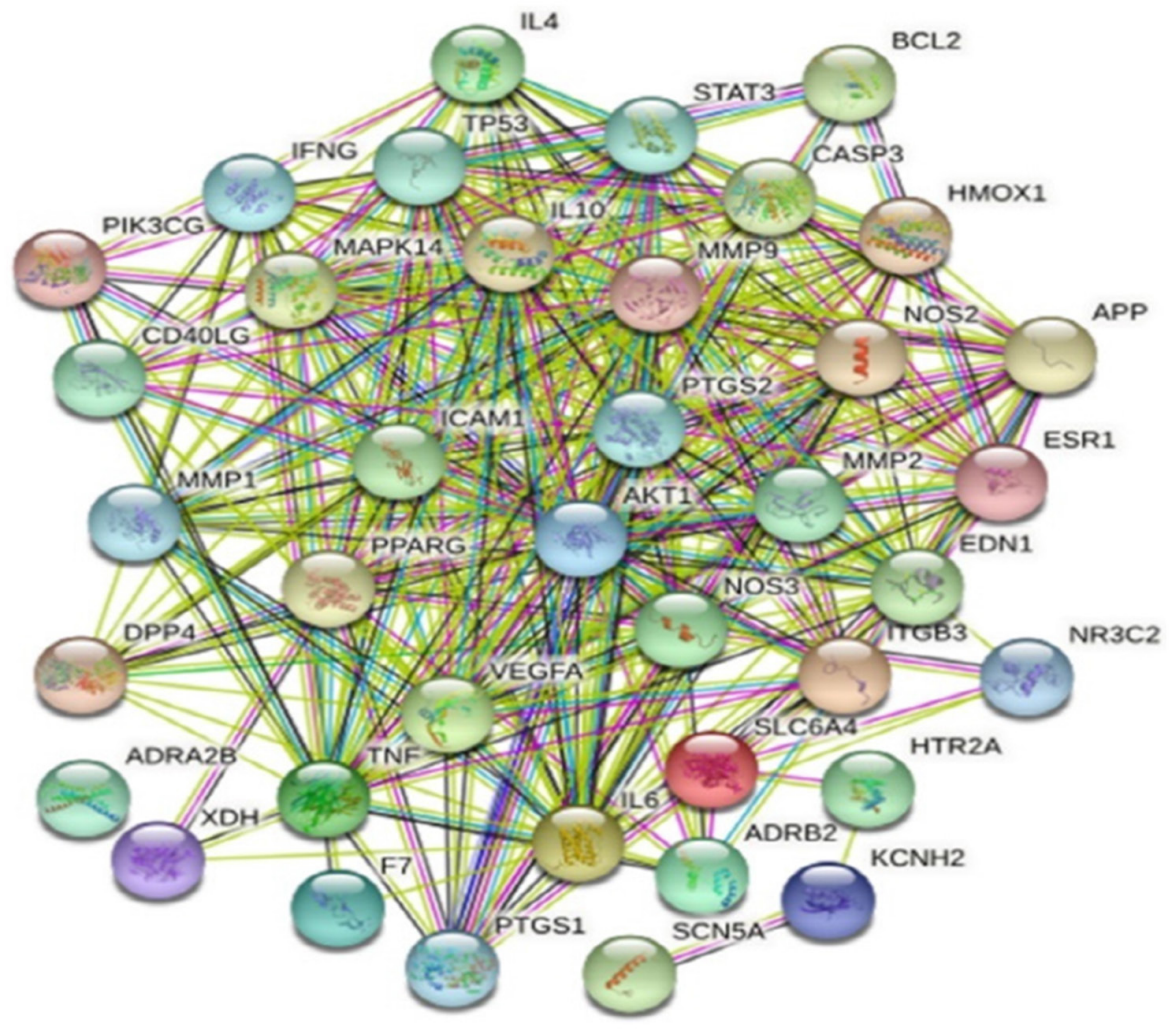
The protein–protein interaction network.

A total of 38 genes were imported into the STRING database for analysis, and the results were shown in Figure 2. The protein–protein interaction network diagram contained 38 nodes and 320 edges with an average node degree of 16.8. The nodes represented proteins, while the edges represent interactions between proteins.

#### 3.1.6 Analysis of gene ontology enrichment of pathways

**FIGURE 3:**
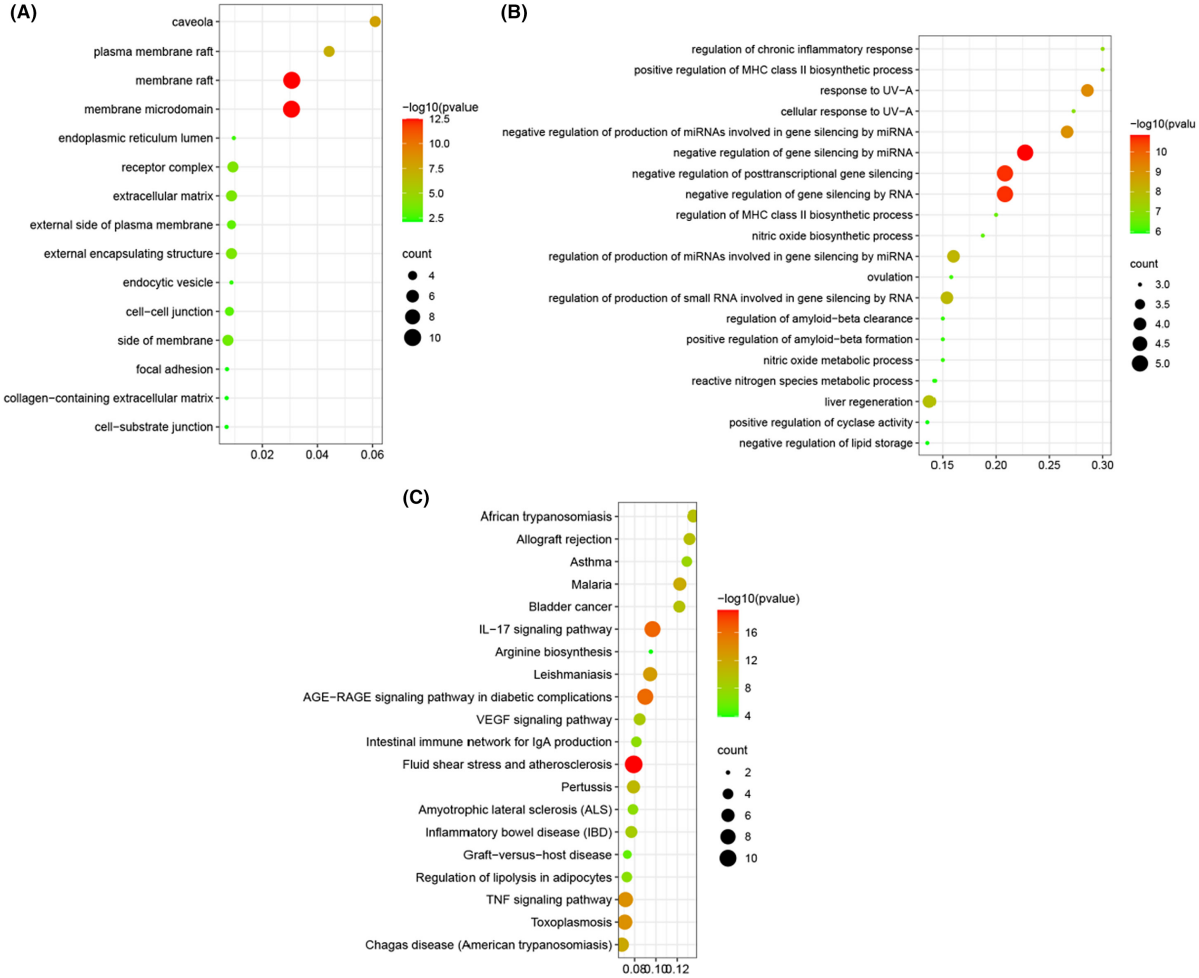
Gene ontology (GO) analyses and Kyoto Encyclopaedia of Genes and Genomes (KEGG) pathway enrichment analyses. (A) GO analyses. The x‐axis represents the counts of the target symbols in each pathway. The y‐axis represents the categories of ‘biological process’ in the GO of the target genes. (B) GO analyses. The x‐axis represents the counts of the target symbols in each pathway. The y‐axis represents the categories of ‘cellular component’ in the GO of the target genes. (C) KEGG pathway enrichment analyses. The x‐axis represents counts of the target symbols in each pathway; The y‐axis represents the categories of ‘KEGG pathway’ in the KEGG of the target genes.

To identify in detail the biological characteristics of putative targets of *S. miltiorrhiza* Bunge on MI, GO and pathway enrichment analysis of the involved targets was conducted via the functional annotation tool in the Metascape database. In total, 175 biological processes, 16 cellular components and 103 molecular functions were obtained. By sequencing, the top 20 most significantly enriched results in the categories of biological processes (BP) and cellular components (CC) were shown in Figure 3A,B. The larger the midpoint in the figure and the darker the colour, the more the input gene sets were enriched in a given pathway and the higher the enrichment significance, respectively. The results indicated that *S. miltiorrhiza* Bunge regulated blood vessel endothelial cell migration, membrane raft and cytokine receptor binding to exert its therapeutic effects in the context of MI. To explore the underlying involved pathways of *S. miltiorrhiza* Bunge on MI, KEGG analysis was performed on 38 targets, and the top 20 most significantly enriched in KEGG are shown in Figure 3C.

#### 3.1.7 Binding capacity between the active ingredients and potential targets by molecular docking

**FIGURE 4:**
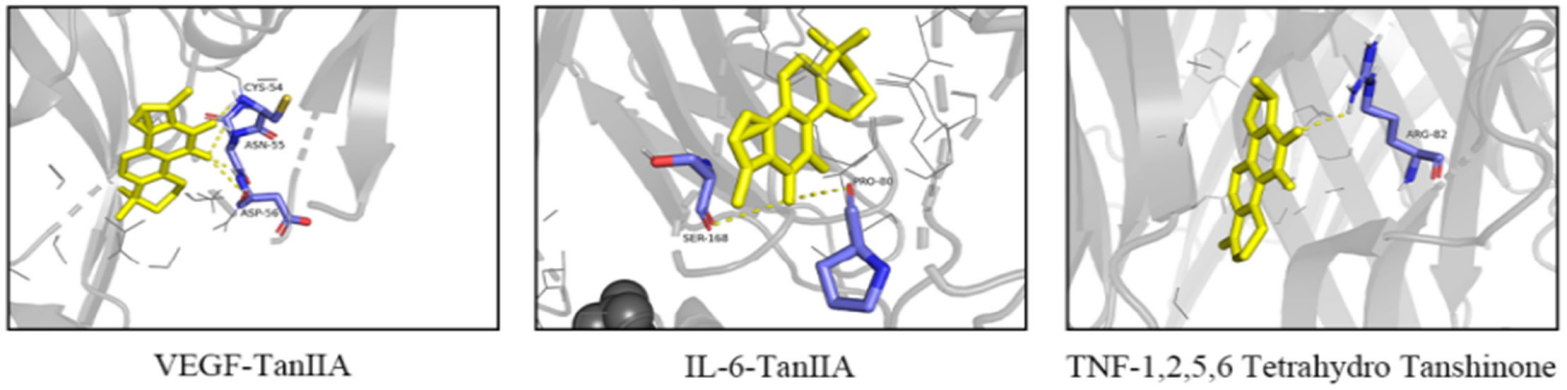
Molecular docking. Binding studies of selected ingredients–targets interactions. Molecules are represented by a ball‐and‐stick model; the hydrogen bonds are represented by a dotted line; and the distance is in angstroms. Atoms C, O and N are purple, blue and red, respectively.

The X‐ray crystal structures of interleukin IL‐6, tumour necrosis factor (TNF), AKT1 and VEGFA were obtained from the RCSB Protein Data Bank (www.rcsb.org); the PDB entry code for these proteins is 4CNI, 2AZ5, 1UNQ and 3QTK, respectively. This was followed by molecular docking with tanshinone IIA, 1, 2, 5, 6‐tetrahydrotanshinone and luteolin. Vina software was used to detect the binding energy of key disease genes and key active ingredients of drugs (Table 2). Lower vina scores indicated a stronger and more stable interaction between the compound and receptor. Among them, the binding conformations of VEGFA, IL‐6, tanshinone IIA, TNF and 1,2,5,6‐tetrahydrotanshinone were found to be more stable, and these were visualized by Pymol (Figure 4). In order to confirm the results by molecular docking, Tanshinone IIA was selected for subsequent in vivo and in vitro verification experiments.

**TABLE 2:** Molecular docking results of the main active components and core targets.

Chemical compound	Binding energy kcal^−1^·mol^−1^			
	IL6	TNF	AKT1	VEGFA
Luteolin	−8.4	−9.0	−6.4	−8.2
Tanshinone IIA	−9.0	−9.0	−7.4	−9.6
1,2,5,6‐tetrahydrotanshinone	−8.6	−9.5	−7.3	−8.7

Abbreviation: TNF; tumour necrosis factor.

### 3.2 Experimental validation

#### 3.2.1 TanIIA protect against myocardial injury and improve cardiac function in LAD mice

**FIGURE 5:**
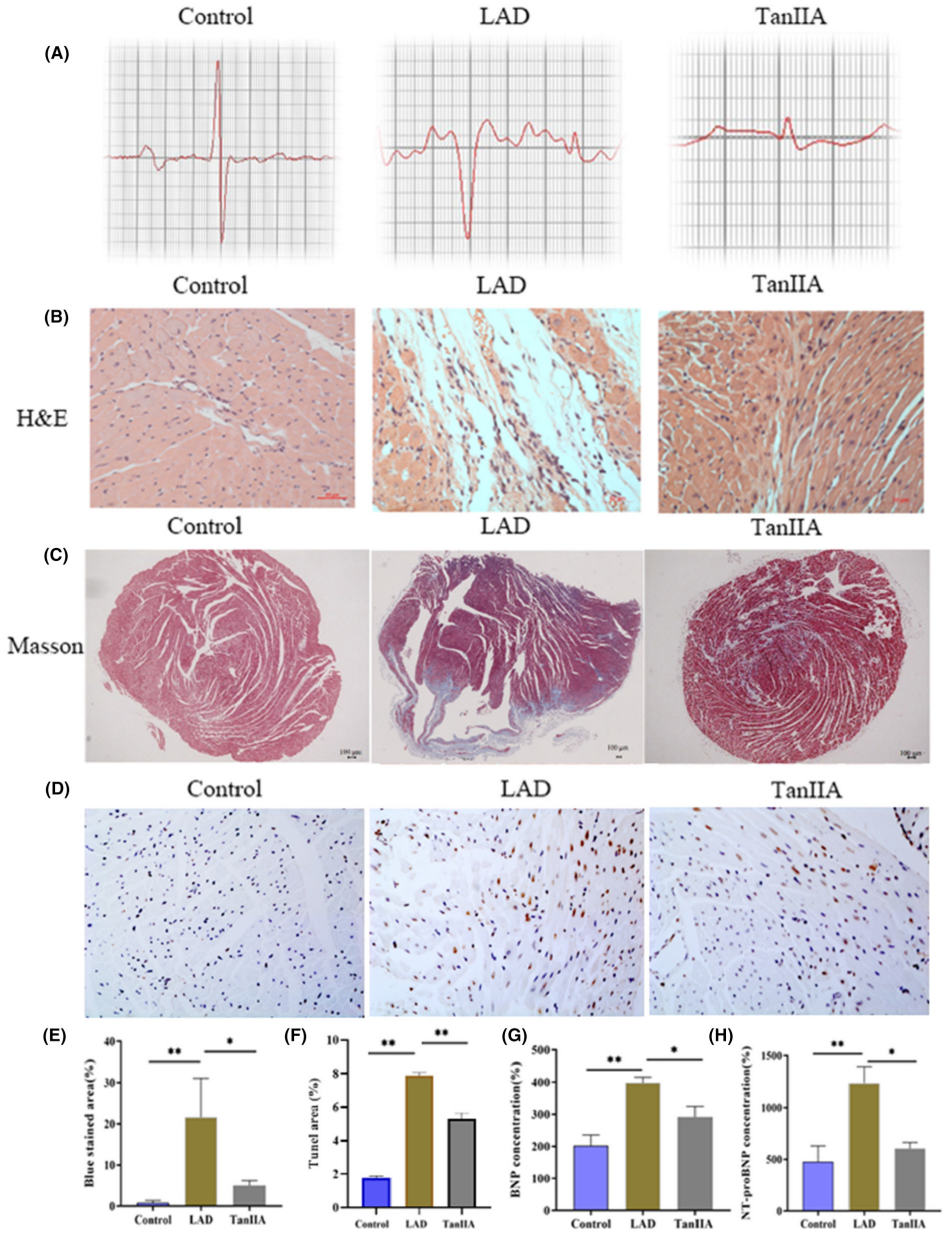
TanIIA alleviates the treatment of myocardial infarction. (A) Representative images of mice electrocardiogram. (B) Haematoxylin and eosin staining. (C, E) Representative images and quantitative analysis of Masson staining. (D, F) Representative images and quantitative analysis of Tunel staining. (G, H) Quantitative analysis of brain natriuretic peptide and N‐terminal pro‐brain natriuretic peptide concentration, and results are expressed as mean ± SD, *n* = 6, **p < 0.001, *p < 0.05.

**FIGURE 6:**
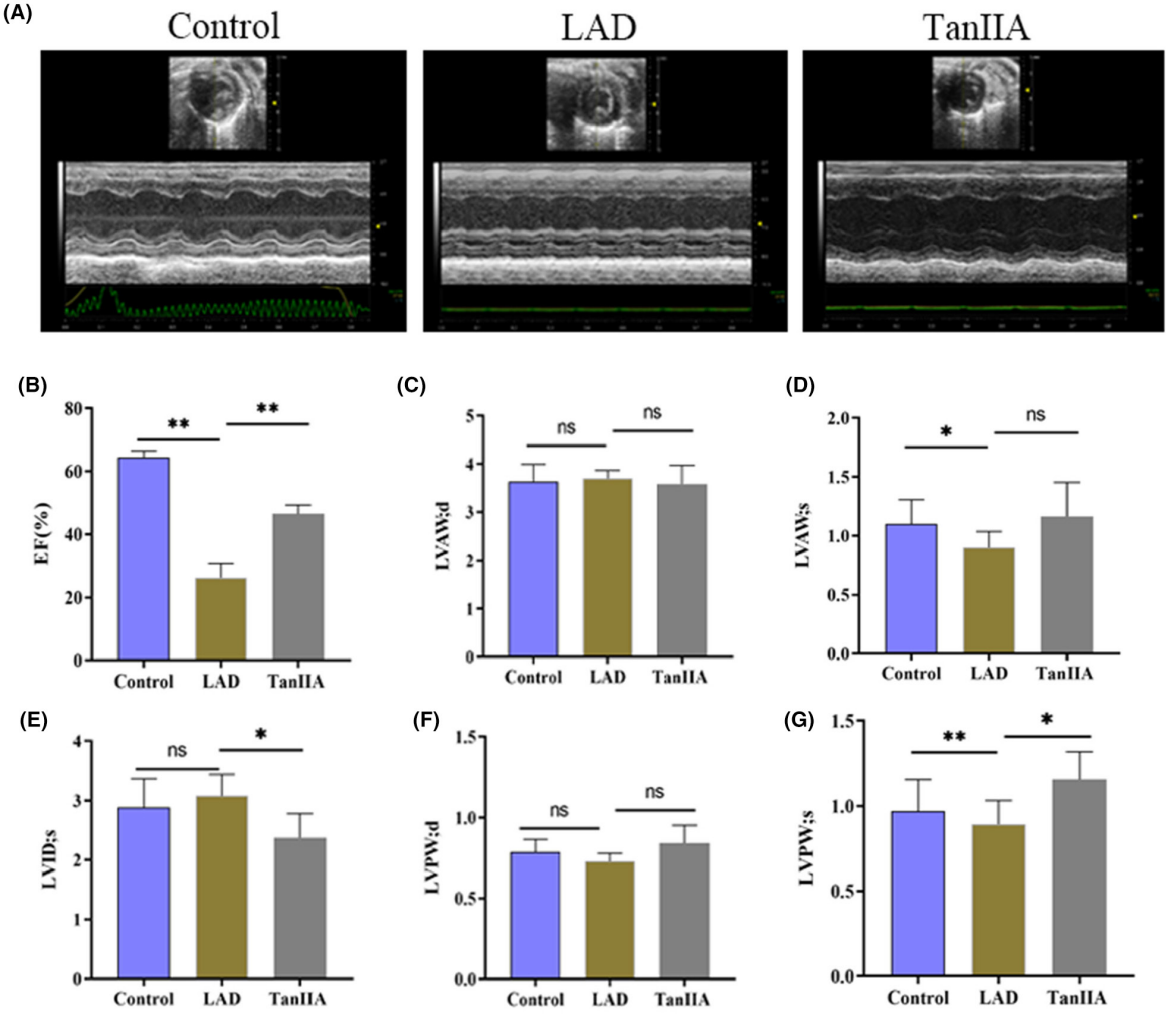
Six weeks after myocardial infarction, echocardiography was performed to evaluate cardiac function. (A) Representative images of mice echocardiography and quantitative analysis of EF (B), LVPW;d (F), LVAW;d (C), LVID;d (E), LVPW;s (G), LVAW;s (D), (mm). Results are expressed as mean ± SD, *n* = 6, **p < 0.001, *p < 0.05, and ns indicates not significant.

According to the electrocardiogram results, the ST‐segment depression in the Tan IIA group was not as significant as in the LAD group (Figure 5A and Figure S1). In the control group, the structure of the myocardium and capillary was intact, the myocardial fibres were regularly arranged, and neither cell swelling nor apparent pathological changes were found. Nevertheless, in the LAD group, the arrangement of myocardial fibres was disordered, the vascular wall was dropsy and the myocardial tissue was faulted. Compared with the LAD group, Tan IIA partially attenuated the myocardial tissue histopathological damage (Figure 5B). The infarction size in the TanIIA group was significantly smaller than that of the LAD group (Figure 5C,E). We speculated that this was due to TanIIA reducing cell apoptosis after modelling. (Figure 5D,F). The results of the Tunel staining demonstrated that the number of apoptotic cardiomyocytes significantly increased in the LAD group. However, after treatment with TanIIA, the number of apoptotic cardiomyocytes showed a decrease. Furthermore, Tan IIA reduced the content of BNP and NT‐proBNP (Figure 5G, H). The results of the echocardiography indicate that EF, LVAW; s and LVPW; s of Tan IIA group were improved compared with the LAD group (Figure 6).

#### 3.2.2 TAN IIA antagonizes oxygen‐glucose deprivation injury and improves endothelial cell function

**FIGURE 7:**
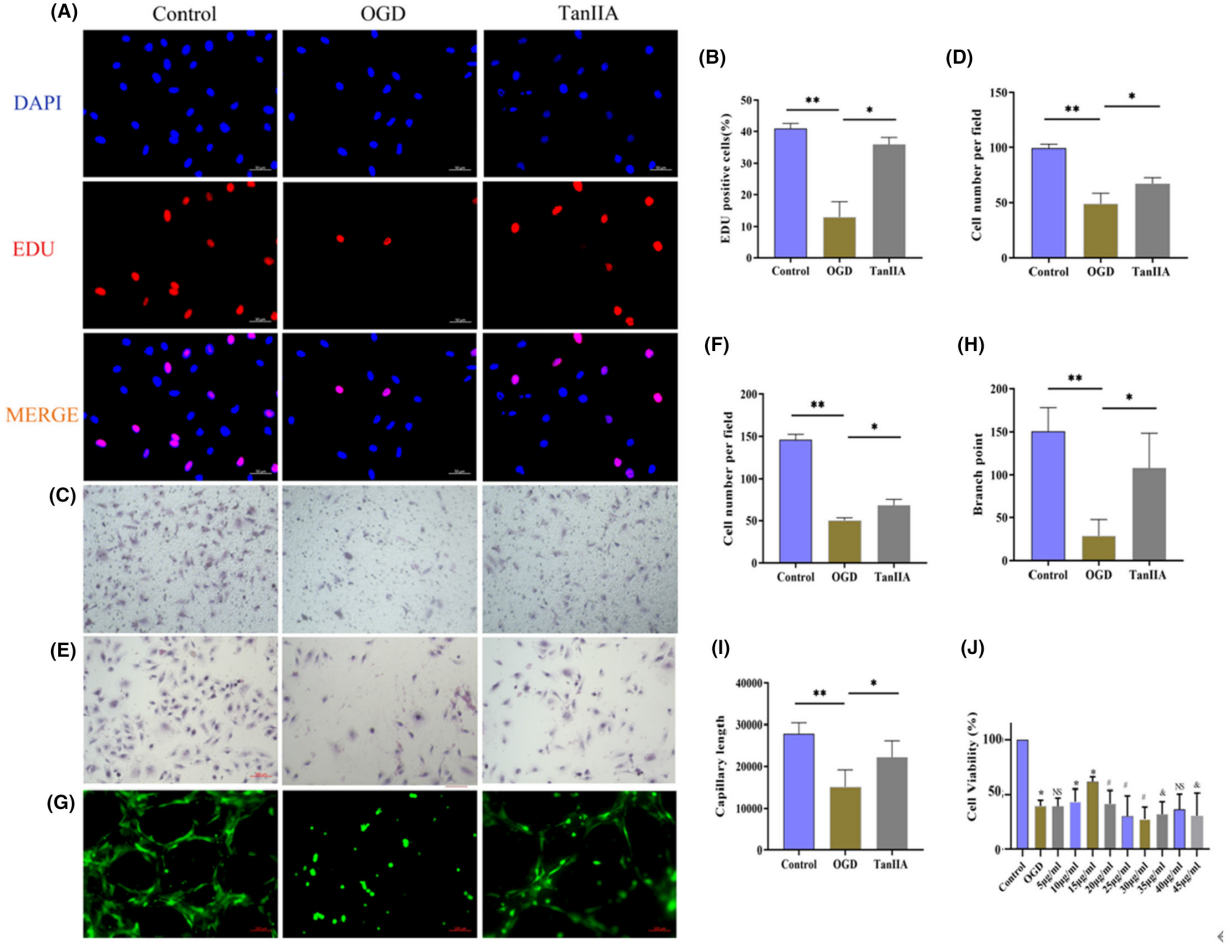
TanIIA affects human umbilical vein endothelial cells (HUVECs) proliferative activity, migration, adhesion and tubule formation. (A, B) Representative images and quantitative analysis of proliferative activity. (C, D) Representative images and quantitative analysis of migration assay (10×). (E, F) Representative images and quantitative analysis of adhesion. (G, H, I) Representative images and quantitative analysis of capillary‐like tubules in HUVECs. (J) Quantitative analysis of CCK8. **p < 0.01, *p < 0.05, ^#^ *p* < 0.05, ^&^ *p* < 0.05.

The DNA replication activity (Figure 7A,E), cell migration (Figure 7B,F), adhesion (Figure 7C,G) and tubular structures (Figure 7D,H) were all reduced in the OGD group. Compared with the OGD group, the DNA replication activity, cell migration and adhesion and the ability to form tubular structures were significantly all increased in the TanIIA group. These results demonstrated that TAN IIA antagonizes oxygen‐glucose deprivation injury and improves endothelial cell function. In addition, the result of CCK8 showed that cell viability decreased after OGD injury and partially recovered after using Tan IIA (Figure 7J). We further evaluated the effect of Tan IIA on vascular endothelial production in vivo. CD31 was used to evaluate the number of endothelial cells in blood vessels, and the results showed that Tan IIA partially restored the damage to endothelial cells caused by LAD. Additionally, new small blood vessels are generated in the infarcted area of the myocardium (Figure S2).

#### 3.2.3 Tan IIA protects mice and human umbilical vein endothelial cells by activating VEGFA and TGF‐β signalling pathways

**FIGURE 8:**
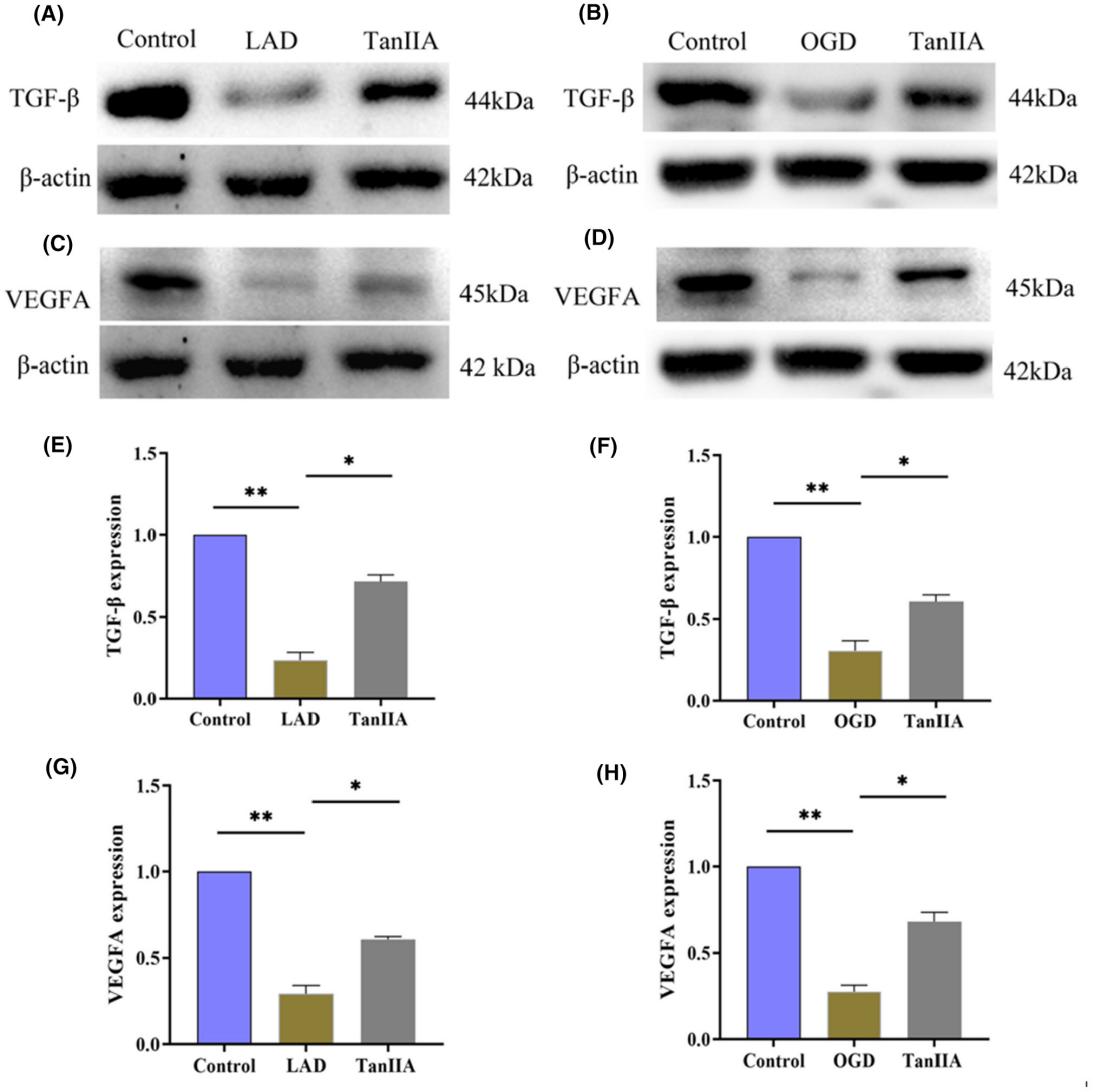
TanIIA treat myocardial infarction by activating VEGFA signalling pathway. Western blot analysis of VEGFA and TGF‐β expression in mice (A, C, E, G). Western blot analysis of VEGFA and TGF‐β expression in human umbilical vein endothelial cells. (B, D, F, H) **p < 0.01, *p < 0.05. Full‐length blots are presented in Figure S1 and Figure S2.

The VEGF and TGF‐β signalling pathways play a major role in the proliferation, migration and sprouting of HUVECs. Therefore, we next detected the expression levels of VEGFA and TGF‐β in both mice and HUVECs. As shown in Figure 8, after the treatment of TanIIA, the expression of both VEGFA and TGF‐β were significantly increased compared with the LAD and OGD group. This suggested that Tan IIA can treat MI by activating the VEGFA and TGF‐β pathways.

## 4 DISCUSSION

Myocardial infarction involves the acute necrosis of the myocardium caused by unstable ischaemic syndrome and is related to various proteins and pathways.^10^ *S. miltiorrhiza* Bunge has the ability to treat MI, but its specific pharmacological mechanism is not fully understood. Therefore, we focused on this point and utilized network pharmacology to elucidate the pharmacological mechanism of *S. miltiorrhiza* Bunge in alleviating MI. In vitro and in vivo experiments were used to verify the reliability of important active ingredients and targets.

Combined with drug target prediction and pathway analysis, *S. miltiorrhiza* Bunge appears to play a role in the treatment of MI by regulating cell migration and proliferation.^11^ Through network pharmacology prediction, we obtained that VEGFA, TNF, HIF‐1 and other pathway targets were most likely to be impacted. The expression of VEGFA in the Tan IIA group was significantly higher than that in the LAD and OGD group. It has been reported that VEGFA in HUVECs could stimulate cell proliferation, cell migration and tubular structure, thereby playing an active role in improving vascular repair.^12^ VEGFA could promote cell viability in infarcted hearts by enhancing cell survival and reducing infarct size in LAD model mice.^13^ In addition to the validation of the VEGFA pathway,^14^ we also tested transforming growth factor‐β (TGF‐β). The results showed that the expression level of TGF‐β in the Tan IIA group was significantly higher than that observed in the LAD and OGD group. Additionally, the in vitro results showed that TanIIA can reduce the area of infarcted myocardium and improve cardiac function. In vivo experiments have shown that Tan IIA can increase cell functions such as proliferation, adhesion and migration. Therefore, our findings suggest that Tan IIA may treat MI by regulating cell migration and proliferation through the VEGFA and TGF‐β signalling pathways. Interestingly, TGF‐β is believed to have a promoting effect on the process of fibrosis and is widely used to induce cell fibrosis.^15^, ^16^ However, TGF‐β is a pleiotropic cytokine with the potential to promote angiogenesis after MI. The experimental results showed that Tan IIA upregulated TGF‐β. We speculate that TGF‐β promote angiogenesis by promoting the proliferation and migration of endothelial cells. The newly formed blood vessels alleviate the damage caused by hypoxia, provide oxygen and nutrients to the myocardium and reduce myocardial cell death. From this perspective, a decrease in myocardial cell death does not require extensive fibrosis to maintain cardiac morphology.

In summary, a comprehensive strategy involving network pharmacological analysis and experimental verification was adopted to determine the potential active components and molecular mechanisms underlying *S. miltiorrhiza* Bunge in the treatment of MI.

## AUTHOR CONTRIBUTIONS

**Xueying Huang:** Data curation (equal); formal analysis (equal); validation (equal); visualization (equal); writing – original draft (equal). **Muxin Zhang:** Writing – original draft (equal). **Yu Song:** Validation (equal). **Bowen Sun:** Formal analysis (equal). **Lin Lin:** Validation (equal). **Xiaoli Song:** Writing – review and editing (equal). **Chao Li:** Writing – review and editing (equal).

## FUNDING INFORMATION

This work was supported by the National Natural Science Foundation of China (grant number 82004276, 82374408).

## CONFLICT OF INTEREST STATEMENT

The authors declare that they have no known competing financial interests or personal relationships that could have appeared to influence the work reported in this paper.

## Supporting information


Figures S1–S2

## Data Availability

The data that support the findings of this study are available on request from the corresponding author (C.L) upon reasonable request.
