# Supplementary material for: Integrated network pharmacology to investigate the mechanism of Salvia miltiorrhiza Bunge in the treatment of myocardial infarction
Source: J Cell Mol Med. 2023 Aug 29;27(22):3514–25. doi: 10.1111/jcmm.17932 (PMC10660626; doi:10.1111/jcmm.17932)
Supplement: Supplementary file 1 — Figures S1–S2 [file JCMM-27-3514-s001.docx]

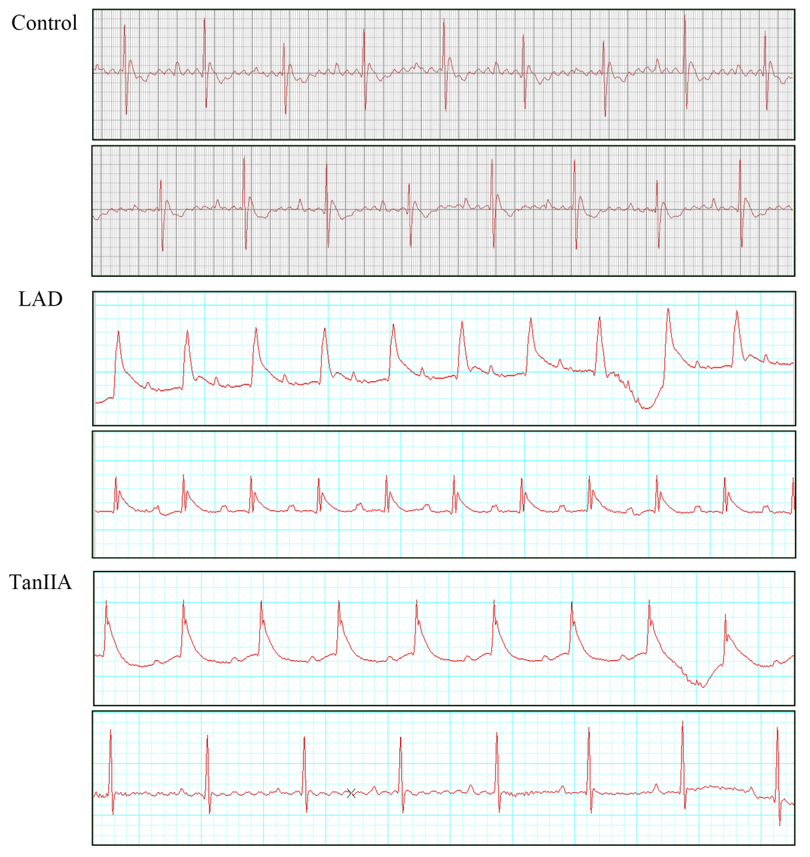


**Supplementary Figure 1** | Representative images of Mice electrocardiogram,


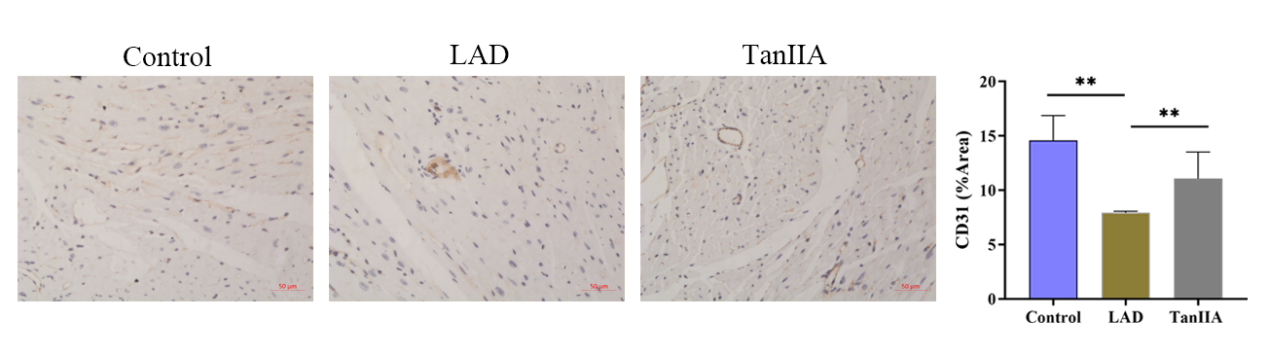


**Supplementary Figure 2** | Representative images of IHC staining of the infarcted myocardium, the brownish region is CD31, results are expressed as mean ±SD, n=6, **P<0.001.
